# Association between CRP-TyG index and hepatic synthetic function in post-myocardial infarction ICU patients

**DOI:** 10.3389/fmed.2025.1710430

**Published:** 2025-12-11

**Authors:** Jiao Bao, Jiejun Hu, Xiangyin Lv

**Affiliations:** 1 Department of Emergency, Affiliated Dongyang Hospital, Wenzhou Medical University, Dongyang, Zhejiang, China; 2 Department of Gastroenterology, Affiliated Dongyang Hospital, Wenzhou Medical University, Dongyang, Zhejiang, China

**Keywords:** CRP-TyG index, hepatic synthetic function, myocardial infarction, intensive care unit, albumin (ALB)

## Abstract

**Objective:**

To determine whether the CRP–TyG index (CTI)—a composite of systemic inflammation and insulin resistance—is independently associated with early hepatic synthetic function in post–myocardial infarction (MI) ICU patients within 24 h.

**Methods:**

Single-center, retrospective cross-sectional study of 286 consecutive post-MI ICU patients (standardized laboratory sampling within 24 h). CTI integrated C-reactive protein with the triglyceride–glucose index. Outcomes were serum albumin (ALB), prealbumin (PA), and the total bilirubin-to-albumin ratio (TBIL/ALB). Associations were estimated using ordinary least squares with prespecified covariate adjustment; nonlinearity was tested using restricted cubic splines. Incremental discrimination was assessed via AUC, net reclassification improvement (NRI), and integrated discrimination improvement (IDI). Prespecified subgroups (sex, age ≤/>70 years) and sensitivity analyses (excluding deaths, pressor users, CRRT) evaluated robustness.

**Results:**

Higher CTI was independently associated with lower ALB (*β* per unit CTI = −1.23 g/L, *p* < 0.001) and PA (*β* = −26.35 mg/L, *p* < 0.001), but not with TBIL/ALB (*β* = 0.02, *p* = 0.135). Compared with Q1, Q4 had lower ALB (−2.19 g/L, *p* = 0.002) and PA (−56.05 mg/L, *p* < 0.001), with linear dose–response supported by splines (ALB *p*-overall <0.001; PA *p*-overall <0.001; nonlinearity *p* > 0.30). Adding CTI improved discrimination for ALB and PA, but not for TBIL/ALB. Effects were larger in males (per-unit: ALB −2.16 g/L; PA −38.92 mg/L) than females (ALB −0.92 g/L; PA −15.87 mg/L). Sensitivity analyses excluding deaths, pressor users, or CRRT yielded consistent estimates.

**Conclusion:**

Within 24 h after myocardial infarction surgery, elevated CTI was associated with decreased albumin and prealbumin levels, but not TBIL/ALB ratio. The associations were the same across sex and age categories.

## 1 Introduction

Myocardial infarction (MI) remains a leading cause of morbidity and mortality worldwide. According to reports from the World Health Organization, cardiovascular diseases accounted for approximately 19.8 million deaths in 2022, representing 32% of the total global mortality (1). Postoperative management in the intensive care unit (ICU) is crucial for patient prognosis, affecting about 48% of patients with acute myocardial infarction (AMI) who require intensive care support (2). The complex pathophysiological processes in postoperative MI patients involve cascades of inflammation, metabolism (3), and organ dysfunction, significantly impacting liver synthetic function (4, 5). Furthermore, postoperative liver synthetic function and liver injury are key determinants of clinical outcomes.

The CRP-TyG index (CTI) is a novel composite biomarker that combines C-reactive protein and triglyceride-glucose index, first proposed by Ruan et al. (6). It has emerged as a robust indicator of systemic inflammation and insulin resistance (7). Increasing evidence suggests that CTI has superior predictive value for cardiovascular diseases compared to single inflammatory or metabolic markers (8, 9). Recent studies have indicated that elevated CTI levels are associated with an increased risk of adverse cardiovascular outcomes and mortality, particularly in patients with acute coronary syndrome, highlighting its potential clinical value in risk stratification (10, 11). The liver’s synthetic function is primarily assessed through serum albumin (ALB), prealbumin (PA), and the bilirubin-albumin ratio, with its protein synthesis capacity directly reflecting overall metabolic homeostasis and the patient’s recovery potential (12, 13).

Although previous studies have confirmed that inflammatory states and metabolic dysfunction can impair hepatic protein synthesis (14, 15), the specific relationship between the comprehensive inflammatory-metabolic burden reflected by CTI and hepatic synthetic capacity in post-myocardial infarction ICU patients remains inadequately characterized. Existing literature primarily focuses on single inflammatory markers such as CRP or metabolic indices like the TyG index, failing to capture the synergistic effects of inflammation and metabolic dysregulation on liver function (8). Furthermore, most studies examining hepatic synthetic function in cardiac patients have concentrated on chronic heart failure populations rather than the acute postoperative ICU setting (16), creating a significant knowledge gap in understanding the immediate hepatic response post-surgery.

This study aims to investigate the association between CTI and key hepatic synthetic function markers (ALB, PA, and TBIL/ALB ratio) within the critical 24 h post-myocardial infarction in ICU patients, validating the hypothesis that elevated CTI levels are independently related to impaired hepatic protein synthesis. The innovation of this research lies in the comprehensive assessment of novel composite biomarkers on the clearly defined acute cardiac ICU population’s hepatic synthetic function, potentially providing clinicians with a more integrated evaluation tool for the early identification of patients at risk for liver dysfunction and adverse clinical outcomes, thereby facilitating targeted therapeutic interventions and improving patient management strategies.

## 2 Methods

### 2.1 Research design and population

This is a single-center, retrospective study employing a cross-sectional analysis framework, focusing on the initial 24 h following admission to the ICU after myocardial infarction (MI) surgery. The study included patients who underwent MI surgery and were admitted to the ICU at a tertiary comprehensive hospital in Zhejiang Province, China, from January 2014 to December 2023.

Inclusion criteria: adult patients (age ≥ 18 years) who were admitted to the ICU following myocardial infarction surgery and had complete laboratory data for C-reactive protein (CRP), fasting triglycerides, fasting blood glucose, serum albumin (ALB), prealbumin (PA), and total bilirubin within 24 h of ICU admission. Exclusion criteria: (1) missing any primary exposure or outcome variables; (2) repeated ICU admissions during the same hospitalization (only the first ICU admission was analyzed); (3) laboratory data obtained outside the 24-h window. All participants were screened for a history of chronic liver disease (e.g., viral hepatitis, cirrhosis, autoimmune liver disease, hepatocellular carcinoma), and none met these criteria; thus, patients with known hepatic dysfunction were excluded by design.

**Figure 1:**
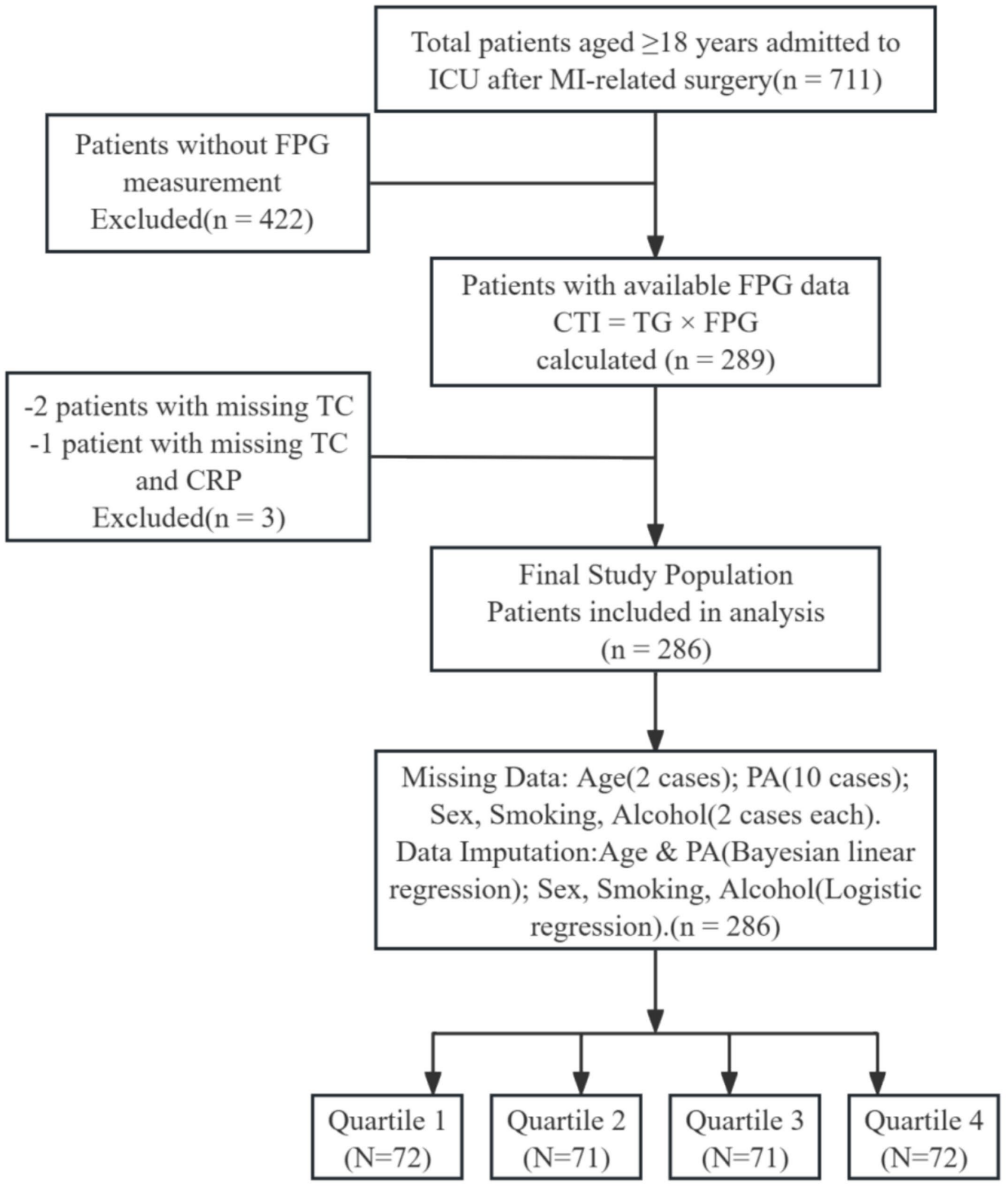
Flowchart of research subject selection.

During the study period, a total of 711 patients who underwent post-myocardial infarction treatment were admitted. Based on established inclusion and exclusion criteria, a total of 286 cases were ultimately identified as meeting the requirements, as detailed in Figure 1. The prior sample size followed the Green’s rule for multiple regression (N ≥ 50 + 8 m), where m represents the number of predictive factors. Given that there are approximately 25 covariates in the fully adjusted model, the minimum required sample size is 250, and our cohort of 286 individuals meets this standard as well as the common rule in OLS models of having “10–15 observations per parameter.” We imputed the missing data. Age and physical activity level were imputed using Bayesian linear regression. Smoking/drinking/gender were imputed using logistic regression.

### 2.2 Data collection and calculation

Data were extracted from electronic medical records and hospital information systems. All laboratory tests were conducted at the hospital’s central laboratory in accordance with standard operating procedures that meet clinical accreditation standards.

The CTI combines CRP and the triglyceride-glucose index (TyG), referencing previous literature and validated by multiple studies; CRP is measured in mg/L using a conventional immunoturbidimetric method; TyG = TG × FPG, with unit conversions performed as necessary to ensure consistency of the formula. The CTI is calculated as follows: CTI = 0.412 × ln[CRP(mg/L)] + ln[TG(mg/dL) × FPG(mg/dL)].

The outcomes measured include liver synthetic function indicators within 24 h: ALB (g/L), PA (mg/L), and the ratio of total bilirubin to albumin (TBIL/ALB, dimensionless), where TBIL is measured in μmol/L and divided by ALB (g/L) to yield a dimensionless ratio.

### 2.3 Covariates

Based on clinical relevance and prior evidence, the following covariates were pre-selected: age, sex, survival status at discharge, smoking, alcohol consumption, hypertension, diabetes mellitus (DM), chronic kidney disease (CKD), congestive heart failure (CHF), chronic obstructive pulmonary disease (COPD), cerebrovascular disease (CVD), malignancy, sepsis, continuous renal replacement therapy (CRRT), vasopressor therapy (dopamine, dobutamine, milrinone, norepinephrine, epinephrine), red blood cell count (RBC), total cholesterol (TC), low-density lipoprotein (LDL), alanine aminotransferase (ALT), aspartate aminotransferase (AST), prothrombin time (PT), blood urea nitrogen (BUN), pre-ICU serum creatinine (Scr before ICU), and maximum serum creatinine after ICU (Max Scr after ICU). Covariate collinearity was assessed prior to model construction (Supplementary Table 1). To further assess the robustness of the observed associations, we performed a sensitivity analysis with additional adjustment for white blood cell count (WBC) as a supplementary inflammation covariate. In the fully adjusted OLS model, WBC count was included as a continuous variable (×10^9^/L).

### 2.4 Statistical analysis

Descriptive statistics summarized the overall characteristics of the patients and the quartile characteristics of CTI: continuous variables were expressed as mean ± standard deviation (SD); categorical variables were presented as counts and percentages. For intergroup comparisons of CTI quartiles, one-way analysis of variance was used for continuous variables, while chi-square tests or Fisher’s exact tests were employed for categorical variables. The primary analysis utilized ordinary least squares (OLS) linear regression, with ALB, PA, and TBIL/ALB as continuous dependent variables, reporting regression coefficients (*β*), standard errors (SE), and 95% confidence intervals (CI). CTI was modeled both continuously (per unit) and by quartiles (with Q1 as the reference). The fully adjusted model controlled for all aforementioned prespecified covariates. As shown in Supplementary Table 1, multicollinearity analysis was conducted for all covariates (with a variance inflation factor, VIF < 10 being considered acceptable). To explore the potential nonlinear relationship between CTI and outcomes, a restricted cubic spline (RCS) function with three knots at the 10th, 50th, and 90th percentiles of CTI was fitted within the OLS framework, and both overall and nonlinear *p*-values were reported. To compare the diagnostic performance of the models, the receiver operating characteristic (ROC) curves and area under the curve (AUC) were calculated before and after adding CTI; the DeLong method was used to compare the AUC, and the net reclassification improvement (NRI) and integrated discrimination improvement (IDI) values for the 95% confidence interval were estimated using the bootstrap resampling method (1,000 iterations). Subgroup analyses were stratified by gender and age (≤70 years vs. >70 years), and the OLS models were repeated. Sensitivity analyses assessed the robustness after excluding cases of mortality, patients receiving only vasopressors, and those undergoing CRRT treatment. As these analyses tested the same underlying hypothesis, no correction for multiple comparisons was applied in the primary presentation. However, Bonferroni-corrected *p*-values are provided in the Supplementary materials to further support the stability of our results. All tests were two-tailed with *α* = 0.05. All analyses were conducted using R (version 4.3.2).

### 2.5 Ethics

This study strictly adheres to the ethical principles outlined in the Declaration of Helsinki. The research protocol has been approved by the hospital's ethics committee (Ethics Approval No: 2025-YX-227). To ensure patient privacy, all clinical data collected during the treatment period have been anonymized. Since this study is a retrospective analysis, all participants signed an informed consent form upon admission, explicitly permitting the use of their clinical data for research purposes.

## 3 Results

### 3.1 Baseline characteristics and distribution of clinical indicators

**Figure 2:**
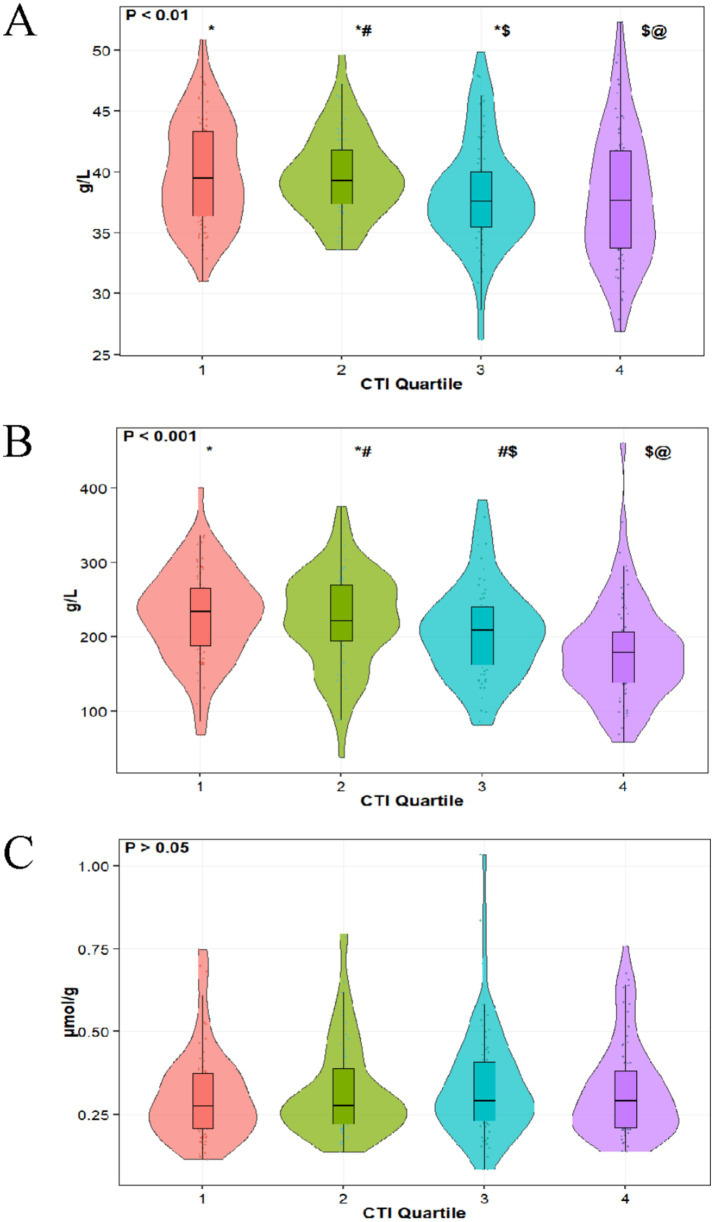
Distribution of ALB **(A)**, PA **(B)**, and TBIL/ALB **(C)** in different CTI quartiles.

This study included 286 patients who underwent coronary artery bypass grafting (CABG) and were admitted to the intensive care unit (ICU), as shown in Table 1. Among them, males accounted for 62.59% (179 cases) and females for 37.41% (107 cases), with an average age of 70.75 ± 12.44 years. The overall mortality rate was 10.49% (30 cases), with 256 patients (89.51%) surviving. Regarding underlying diseases, patients with hypertension comprised 70.28% (201 cases), those with diabetes accounted for 54.20% (155 cases), cardiovascular disease patients made up 31.82% (91 cases), and chronic kidney disease patients constituted 7.34% (21 cases). A history of smoking was reported in 129 cases (45.42%), while a history of alcohol consumption was noted in 95 cases (33.45%). In terms of clinical treatment, 87.76% (251 cases) of patients received vasoactive drug therapy, 60.49% (173 cases) developed sepsis, and 9.09% (26 cases) underwent continuous renal replacement therapy. After grouping by CTI quartiles, significant differences were observed among groups in terms of survival status, diabetes, chronic kidney disease, cardiovascular disease, and the incidence of sepsis (*p* < 0.05). In terms of biochemical indicators, the total cholesterol level was 4.71 ± 1.49 mmol/L, alanine aminotransferase was 37.28 ± 43.75 U/L, prothrombin time was 13.52 ± 1.48 s, and blood urea nitrogen was 6.89 ± 3.98 mmol/L. Among key nutritional indicators, the serum albumin level was 38.77 ± 4.80 g/L, prealbumin level was 210.63 ± 70.09 mg/L, and the ratio of total bilirubin to albumin was 0.33 ± 0.17. With the increase of CTI quartiles, the serum albumin level showed a downward trend (Q1: 39.88 ± 4.27 vs. Q4: 37.58 ± 5.83 g/L, *p* = 0.003), and the prealbumin level also exhibited a significant declining trend (Q1: 234.03 ± 68.64 vs. Q4: 178.34 ± 73.32 mg/L, *p* < 0.001), while there was no significant difference in the ratio of total bilirubin to albumin among the quartiles (*p* = 0.536). Figure 2 illustrates the distribution of ALB, PA, and TBIL/ALB with the increase of CTI quartiles.

**Table 1:** Basic characteristic.

Variable	Overall	1	2	3	4	*p*
	*N* = 286	*N* = 72	*N* = 71	*N* = 71	*N* = 72	
Gender (%)						
Female	107 (37.41)	26 (36.11)	28 (39.44)	23 (32.39)	30 (41.67)	0.683
Male	179 (62.59)	46 (63.89)	43 (60.56)	48 (67.61)	42 (58.33)	
Status (%)						
Survival	256 (89.51)	71 (98.61)	64 (90.14)	65 (91.55)	56 (77.78)	0.001*
Death	30 (10.49)	1 (1.39)	7 (9.86)	6 (8.45)	16 (22.22)	
Smoking (%)						
No	155 (54.58)	44 (61.11)	35 (49.30)	37 (52.86)	39 (54.93)	0.547
Yes	129 (45.42)	28 (38.89)	36 (50.70)	33 (47.14)	32 (45.07)	
Alcohol (%)						
No	189 (66.55)	53 (73.61)	50 (70.42)	39 (55.71)	47 (66.20)	0.123
Yes	95 (33.45)	19 (26.39)	21 (29.58)	31 (44.29)	24 (33.80)	
Hypertension (%)						
No	85 (29.72)	19 (26.39)	21 (29.58)	22 (30.99)	23 (31.94)	0.895
Yes	201 (70.28)	53 (73.61)	50 (70.42)	49 (69.01)	49 (68.06)	
DM (%)						
No	131 (45.80)	39(54.17)	36 (50.70)	33 (46.48)	23 (31.94)	0.040*
Yes	155(54.20)	33(45.83)	35 (49.30)	38 (53.52)	49 (68.06)	
CKD (%)						
No	265 (92.66)	62 (86.11)	70 (98.59)	65 (91.55)	68 (94.44)	0.034*
Yes	21 (7.34)	10 (13.89)	1 (1.41)	6 (8.45)	4 (5.56)	
CHF (%)						
No	213 (74.48)	54 (75.00)	57 (80.28)	49 (69.01)	53 (73.61)	0.491
Yes	73 (25.52)	18 (25.00)	14 (19.72)	22 (30.99)	19 (26.39)	
COPD (%)						
No	248 (86.71)	64 (88.89)	63 (88.73)	59 (83.10)	62 (86.11)	0.711
Yes	38 (13.29)	8 (11.11)	8 (11.27)	12 (16.90)	10 (13.89)	
CVD (%)						
No	195 (68.18)	39 (54.17)	53 (74.65)	58 (81.69)	45 (62.50)	0.002*
Yes	91 (31.82)	33 (45.83)	18 (25.35)	13 (18.31)	27 (37.50)	
Tumor (%)						
No	256 (89.51)	64 (88.89)	64 (90.14)	66 (92.96)	62 (86.11)	0.605
Yes	30 (10.49)	8 (11.11)	7 (9.86)	5 (7.04)	10 (13.89)	
Sepsis (%)						
No	113 (39.51)	27 (37.50)	36 (50.70)	31 (43.66)	19 (26.39)	0.023*
Yes	173 (60.49)	45 (62.50)	35 (49.30)	40 (56.34)	53 (73.61)	
CRRT (%)						
No	260 (90.91)	65 (90.28)	65 (91.55)	63 (88.73)	67 (93.06)	0.831
Yes	26 (9.09)	7 (9.72)	6 (8.45)	8 (11.27)	5 (6.94)	
Vasoactive drug therapy (%)						
No	35 (12.24)	9 (12.50)	9 (12.68)	10 (14.08)	7 (9.72)	0.881
Yes	251 (87.76)	63 (87.50)	62 (87.32)	61 (85.92)	65 (90.28)	
Age [mean (SD)]	70.75 (12.44)	71.47 (11.50)	69.75 (13.89)	71.28 (12.39)	70.49 (12.05)	0.835
RBC [mean (SD)]	4.40 (0.69)	4.39 (0.61)	4.58 (0.62)	4.35 (0.80)	4.29 (0.69)	0.078
TC [mean (SD)]	4.71 (1.49)	4.39 (1.15)	4.68 (1.04)	4.64 (1.30)	5.15 (2.13)	0.020*
HDL [mean (SD)]	0.98 (0.26)	1.02 (0.26)	1.02 (0.26)	0.95 (0.26)	0.94 (0.27)	0.102
LDL [mean (SD)]	2.82 (0.99)	2.74 (1.02)	2.87 (0.77)	2.70 (0.95)	2.98 (1.16)	0.307
ALT [mean (SD)]	37.28 (43.75)	26.54 (23.85)	39.14 (34.87)	36.16 (32.75)	47.29 (68.07)	0.040*
AST [mean (SD)]	92.83 (134.41)	71.93 (123.55)	109.17 (136.14)	91.73 (124.90)	98.72 (151.17)	0.404
PT [mean (SD)]	13.52 (1.48)	13.40 (0.98)	13.28 (0.87)	13.37 (0.89)	14.02 (2.44)	0.009*
BUN [mean (SD)]	6.89 (3.98)	5.93 (2.19)	6.61 (3.00)	6.96 (3.12)	8.03 (6.16)	0.014*
Scr before ICU [mean (SD)]	74.99 (53.18)	67.83 (38.76)	63.32 (19.93)	79.90 (40.81)	88.81 (86.09)	0.016*
pScr_7d [mean (SD)]	161.80 (141.12)	164.79 (168.74)	139.93 (118.82)	168.68 (126.61)	173.60 (145.16)	0.494
ALB [mean (SD)]	38.77 (4.80)	39.88 (4.27)	39.74 (3.39)	37.91 (4.98)	37.58 (5.83)	0.003*
PA [mean (SD)]	210.63 (70.09)	234.03 (68.64)	223.44 (61.17)	206.85 (64.90)	178.34 (73.32)	<0.001*
TBIL/ALB [mean (SD)]	0.33 (0.17)	0.31 (0.13)	0.32 (0.14)	0.34 (0.16)	0.34 (0.21)	0.536

*p < 0.05.

### 3.2 Correlation between CTI and liver synthetic function

**Figure 3:**
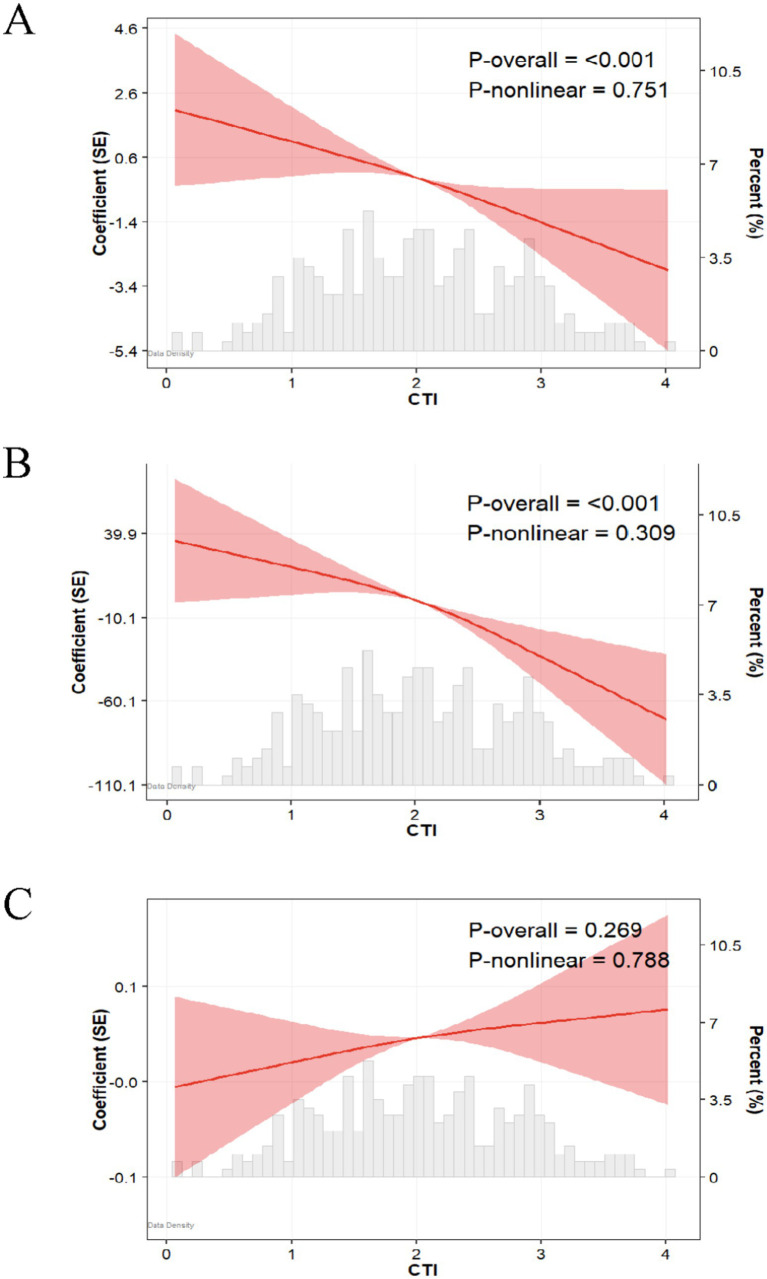
Associations between CTI and ALB **(A)**, PA **(B)**, and TBIL/ALB **(C)** in patients after MI surgery: restricted cubic spline analysis.

**Figure 4:**
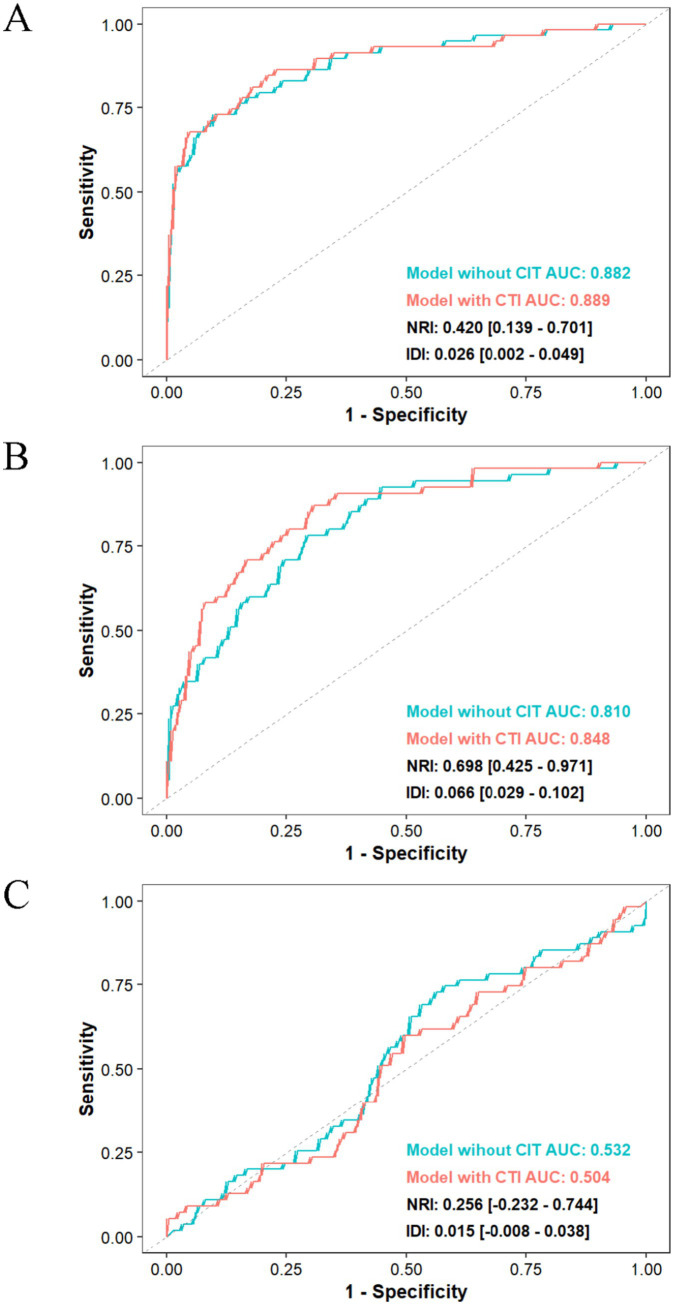
Comparison of the risk discrimination ability of **(A)** serum albumin (ALB), **(B)** prealbumin (PA), and **(C)** the total bilirubin-to-albumin ratio (TBIL/ALB) before and after the inclusion of the CRP–TyG index (CTI) in the model.

As shown in Table 2, this study employed an ordinary least squares (OLS) regression model to systematically evaluate the association between the CRP-TyG index (CTI) and nutritional indicators, using serum albumin (ALB), prealbumin (PA), and the total bilirubin/albumin ratio (TBIL/ALB) as continuous dependent variables. After adequately adjusting for confounding factors, a one-unit increase in CTI was significantly associated with a decrease in serum albumin levels by 1.23 g/L (*β* = −1.23, SE = 0.32, *p* < 0.001) and a decrease in prealbumin levels by 26.35 mg/L (*β* = −26.35, SE = 5.07, *p* < 0.001), while the change in the total bilirubin/albumin ratio was not statistically significant (*β* = 0.02, SE = 0.01, *p* = 0.135). Group analysis based on CTI quartiles indicated that, compared to the Q1 group, serum albumin levels in the Q3 group decreased by 1.81 g/L (*p* = 0.008) and by 2.19 g/L in the Q4 group (*p* = 0.002), with a trend test *p* < 0.001; the prealbumin levels in the Q3 group decreased by 29.48 mg/L (*p* = 0.005) and by 56.05 mg/L in the Q4 group (*p* < 0.001), with a trend test *p* < 0.001; however, there were no statistically significant differences in the total bilirubin/albumin ratio among the quartiles (trend test *p* = 0.081). Logistic regression analysis was performed as a supplementary analysis (Supplementary Table 2). The restrictive cubic spline analysis further confirmed a significant linear negative correlation between the CTI and serum albumin (*p*-overall<0.001, *p*-nonlinear = 0.751) as well as prealbumin (*p*-overall<0.001, *p*-nonlinear = 0.309), while no significant association was found with the total bilirubin/albumin ratio (*p*-overall = 0.269) (Figure 3). ROC curve analysis revealed that after incorporating the CTI, the AUC of the serum albumin model increased from 0.882 to 0.889, with a net reclassification improvement index (NRI) of 0.420 [0.139–0.701] and an integrated discrimination improvement index (IDI) of 0.026 [0.002–0.049]; the AUC of the prealbumin model rose from 0.810 to 0.848, with an NRI of 0.698 [0.425–0.971] and an IDI of 0.066 [0.029–0.102]; however, the predictive ability of the total bilirubin/albumin ratio model did not show significant improvement (AUC decreased from 0.532 to 0.504) (Figure 4).

**Table 2:** Association between the CTI and ALB, PA, TBIL/ALB by OLS model.

Characteristic	ALB-crude		ALB-adjusted	
	*β* (SE)	*p*	*β* (SE)	*p*
CTI (per 1 unit)	−1.21 (0.35)	<0.001*	−1.23 (0.32)	<0.001*
CTI quartile				
Q1	Ref		Ref	
Q2	−0.14 (0.79)	0.863	−0.30 (0.68)	0.66
Q3	−1.97 (0.79)	0.013*	−1.81 (0.67)	0.008*
Q4	−2.30 (0.78)	0.004*	−2.19 (0.71)	0.002*
*p* for trends		<0.001*		<0.001*

Characteristic	PA-crude		PA-adjusted	
	*β* (SE)	*p*	*β* (SE)	*p*
CTI (per 1 unit)	−22.67 (5.01)	<0.001*	−26.35 (5.07)	<0.001*
CTI quartile				
Q1	Ref		Ref	
Q2	−10.01 (11.18)	0.371	−8.56 (10.48)	0.415
Q3	−27.56 (11.18)	0.014*	−29.48 (10.46)	0.005*
Q4	−53.74 (11.14)	<0.001*	−56.05 (10.98)	<0.001*
*p* for trends		<0.001*		<0.001*

Characteristic	TBIL/ABL-crude		TBIL/ABL-adjusted	
	*β* (SE)	*p*	*β* (SE)	*p*
CTI (per 1 unit)	0.02 (0.01)	0.219	0.02 (0.01)	0.135
CTI quartile				
Q1	Ref		Ref	
Q2	0.02 (0.03)	0.571	−0.00 (0.03)	0.995
Q3	0.03 (0.03)	0.263	0.03 (0.03)	0.324
Q4	0.04 (0.03)	0.179	0.04 (0.03)	0.132
*p* for trends		0.146		0.081

*p < 0.05.

Adjusted for age, gender, status, smoking, alcohol, hypertension, diabetes mellitus (DM), chronic kidney disease (CKD), congestive heart failure (CHF), chronic obstructive pulmonary disease (COPD), cardiovascular disease (CVD), tumor, sepsis, continuous renal replacement therapy (CRRT), vasoactive drug use, red blood cell count (RBC), total cholesterol (TC), low-density lipoprotein (LDL), alanine aminotransferase (ALT), aspartate aminotransferase (AST), prothrombin time (PT), blood urea nitrogen (BUN), serum creatinine before ICU (Scr before ICU), and maximum serum creatinine after ICU (Max Scr after ICU).

### 3.3 Gender and age subgroup analysis

**Figure 5:**
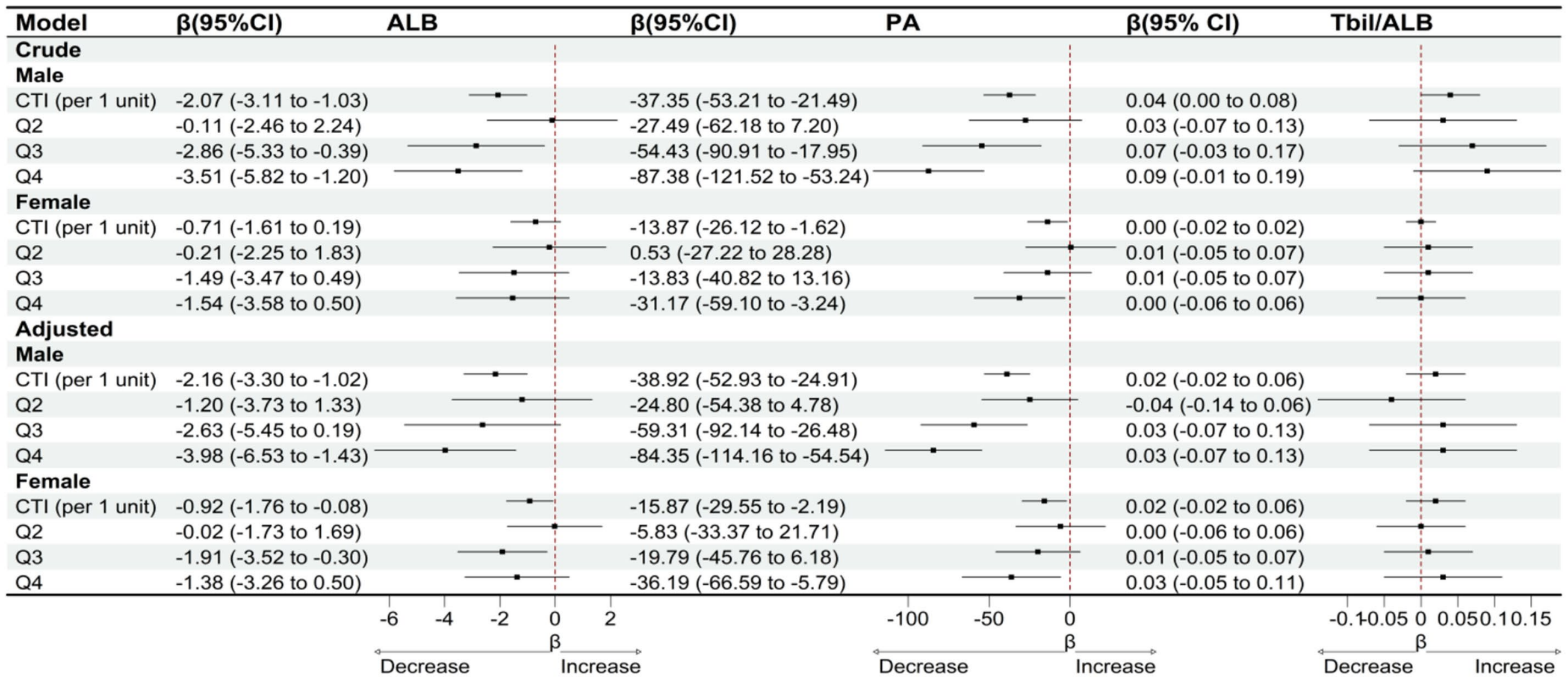
OLS-based associations of CTI with ALB, PA, and TBIL/ALB by gender.

**Figure 6:**
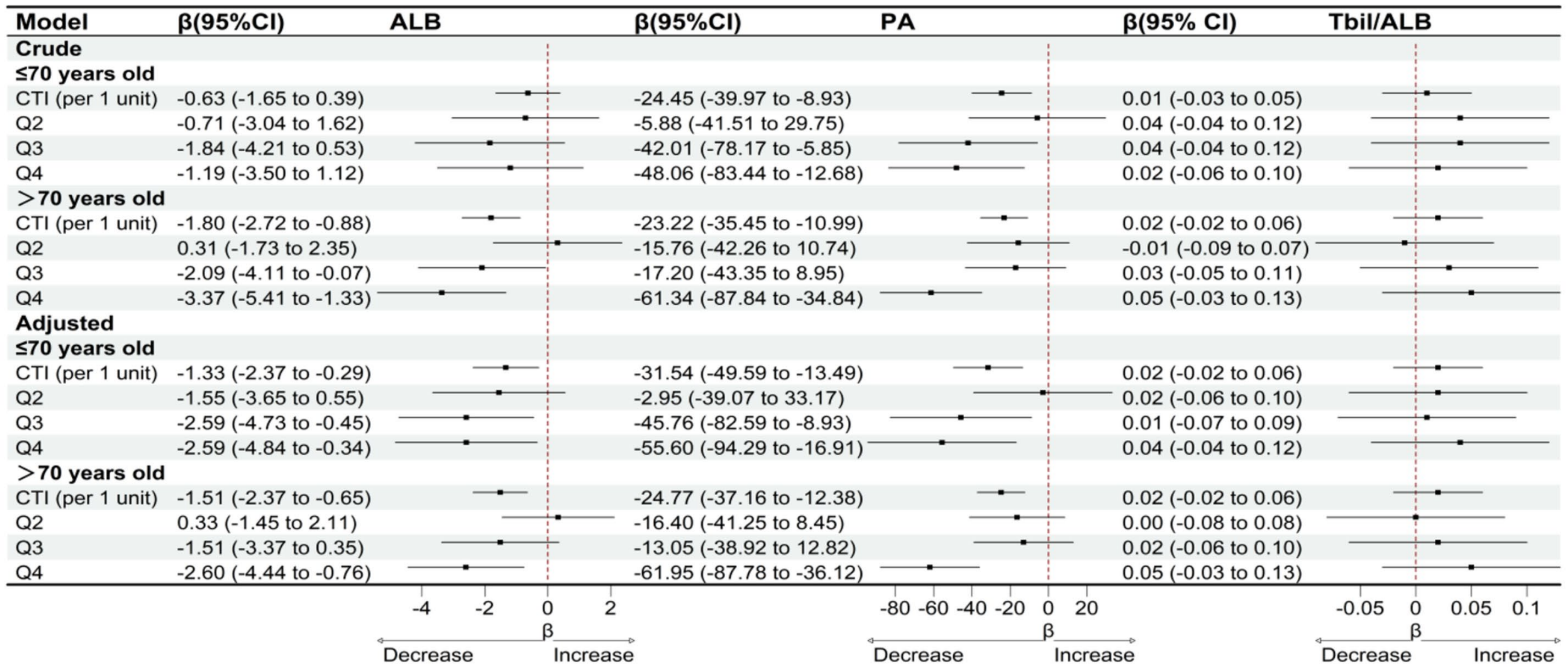
OLS-based associations of CTI with ALB, PA, and TBIL/ALB by age.

Subgroup analyses stratified by gender and age indicate that the association between CTI and clinical liver indicators varies across different populations. In the gender subgroup analysis, as shown in Figure 5, after adjusting for confounding factors, for male patients, with each unit increase in CTI, serum albumin significantly decreased by 2.16 g/L (95% CI: −3.30 to −1.02), and prealbumin significantly decreased by 38.92 mg/L (95% CI: −52.93 to −24.91). However, the change in the total bilirubin/albumin ratio was not statistically significant (*β* = 0.02, 95% CI: −0.02 to 0.06). In female patients, the association between CTI and serum albumin was relatively weaker (*β* = −0.92, 95% CI: −1.76 to −0.08), while a significant negative correlation with prealbumin persisted (*β* = −15.87, 95% CI: −29.55 to −2.19), and the total bilirubin/albumin ratio also showed no significant change (*β* = 0.02, 95% CI: −0.02 to 0.06). Stratified by CTI quartiles, male patients in the Q4 group experienced a decrease in serum albumin by 3.98 g/L (95% CI: −6.53 to −1.43) and a decrease in prealbumin by 84.35 mg/L (95% CI: −114.16 to −54.54), while female patients only showed a significant decrease in serum albumin in the Q3 group (*β* = −1.91, 95% CI: −3.52 to −0.30), with a decrease in prealbumin of 36.19 mg/L (95% CI: −66.59 to −5.79) in the Q4 group. In the age subgroup analysis, as shown in Figure 6, after adjusting for confounding factors, for the age group ≤70 years, for each unit increase in CTI, serum albumin decreased by 1.33 g/L (95% CI: −2.37 to −0.29) and prealbumin decreased by 31.54 mg/L (95% CI: −49.59 to −13.49). In the age group >70 years, for each unit increase in CTI, serum albumin decreased by 1.51 g/L (95% CI: −2.37 to −0.65) and prealbumin decreased by 24.77 mg/L (95% CI: −37.16 to −12.38). Quartile analysis showed that both age groups exhibited the most significant decline in nutritional indicators in the Q4 group, where the serum albumin in the ≤70 years group decreased by 2.59 g/L (95% CI: −4.84 to −0.34) and prealbumin decreased by 55.60 mg/L (95% CI: −94.29 to −16.91). In the >70 years group, serum albumin in Q4 decreased by 2.60 g/L (95% CI: −4.44 to −0.76) and prealbumin decreased by 61.95 mg/L (95% CI: −87.78 to −36.12). However, the total bilirubin/albumin ratio did not show a significant association with CTI in any of the subgroups.

### 3.4 Sensitivity analysis

**Figure 7:**
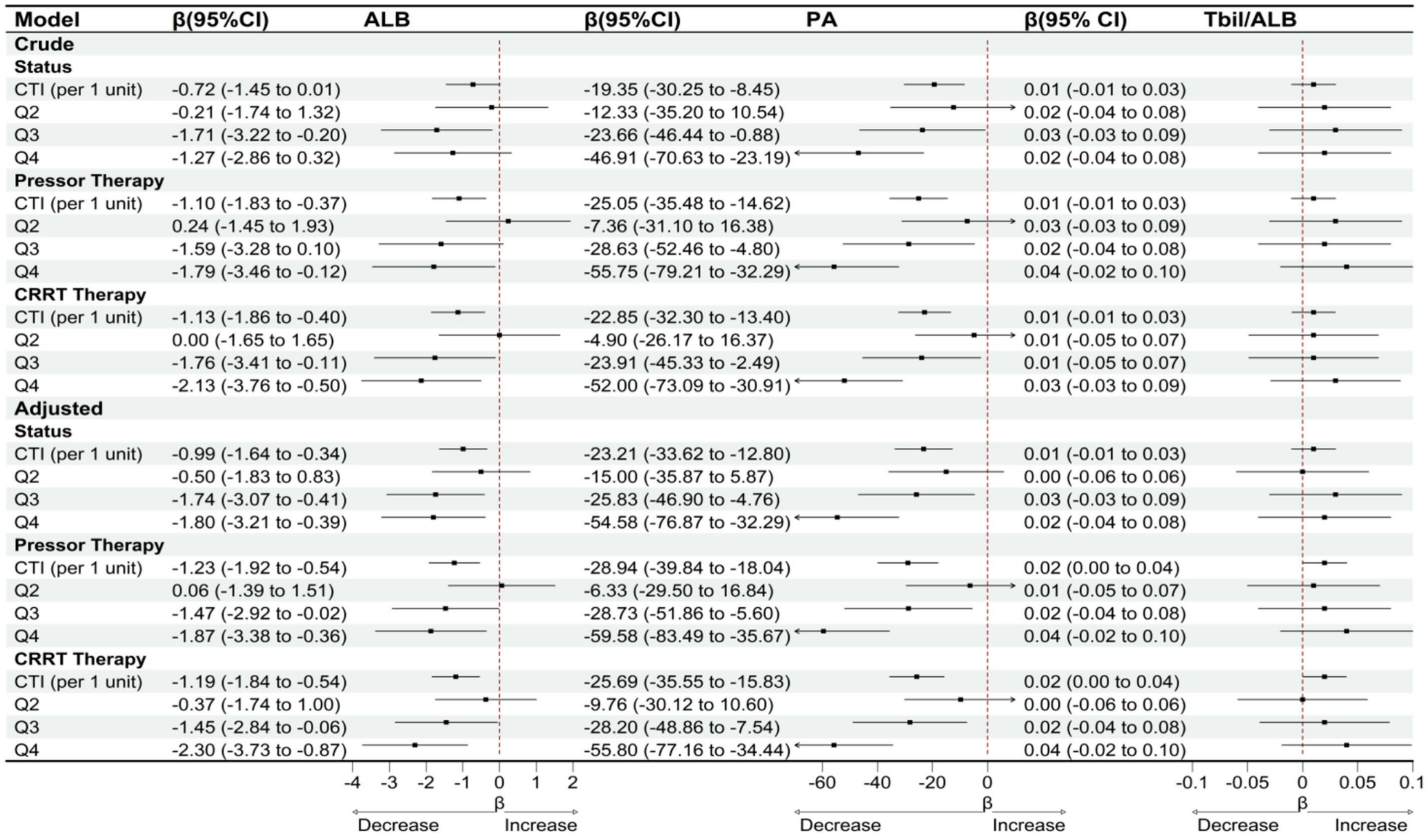
OLS-based associations of CTI with ALB, PA, and TBIL/ALB among patients with status = survival/vasoactive drug therapy/CRRT therapy.

Sensitivity analysis results demonstrated robust stability in the association between CTI and nutritional indicators across various model assumptions and patient subgroups (Figure 7). When patients who died during hospitalization were excluded from the analysis, each unit increase in CTI remained significantly associated with a decline in serum albumin of 0.99 g/L (95% CI: −1.64 to −0.34) and a decrease in prealbumin of 23.21 mg/L (95% CI: −33.62 to −12.80), while the total bilirubin-to-albumin ratio showed no significant change (*β* = 0.01, 95% CI: −0.01 to 0.03). Among patients receiving vasoactive drug therapy, the negative correlation between CTI and both serum albumin (*β* = −1.23, 95% CI: −1.92 to −0.54) and prealbumin (*β* = −28.94, 95% CI: −39.84 to −18.04) was further strengthened. Similarly, in patients undergoing continuous renal replacement therapy (CRRT), the associations between CTI and serum albumin (*β* = −1.19, 95% CI: −1.84 to −0.54) and prealbumin (*β* = −25.69, 95% CI: −35.55 to −15.83) remained statistically significant. Sensitivity analysis stratified by CTI quartiles revealed that the highest quartile (Q4) consistently exhibited the most pronounced decreases in both serum albumin and prealbumin levels across different model specifications. The magnitude of prealbumin decline was most substantial in the Q4 group among patients receiving vasoactive drug therapy (*β* = −59.58, 95% CI: −83.49 to −35.67). Throughout all sensitivity analyses, the total bilirubin-to-albumin ratio failed to demonstrate any significant association with CTI. To further evaluate potential residual confounding by general inflammatory burden, we repeated all primary analyses with additional adjustment for total white blood cell (WBC) count. As shown in Supplementary Table S3, after adjusting for WBC, the associations between CTI and both ALB and PA remained statistically significant and directionally consistent. TBIL/ALB association observed in the primary analysis became non-significant after WBC adjustment (Q4 vs. Q1: *β* = 0.0029, *p* = 0.191).

## 4 Discussion

In this single-center cohort study, we enrolled 286 patients who underwent assessment within 24 h of ICU admission following myocardial infarction (MI). Our findings demonstrate that elevated CRP-TyG index (CTI), reflecting the combined burden of systemic inflammation and insulin resistance, exhibits an independent and linear negative association with hepatic synthetic function. This relationship manifested through decreased serum albumin (ALB) and prealbumin (PA) levels, though no corresponding effect was observed on the total bilirubin-to-albumin ratio (TBIL/ALB). After comprehensive covariate adjustment, each unit increase in CTI corresponded to a 1.23 g/L decrease in ALB and a 26.35 mg/L reduction in PA. Restricted cubic spline analysis supported a linear dose–response relationship between these variables. While CTI provided limited incremental discriminatory capacity to the baseline model at the AUC level, reclassification metrics (NRI/IDI) for both ALB and PA showed improvement, suggesting enhanced granularity in prognostic discrimination. Subgroup analysis revealed consistent negative correlations across different age groups and sexes, with a more pronounced effect observed in male patients. Multiple sensitivity analyses yielded comparable results when excluding deceased patients, those receiving vasoactive drug therapy, and patients undergoing continuous renal replacement therapy (CRRT). Clinically, these findings suggest that CTI may serve as an early composite indicator of impaired hepatic protein synthesis within the immediate post-operative ICU timeframe, potentially facilitating risk stratification and targeted optimization of hemodynamic and metabolic parameters.

Our findings demonstrate biological consistency with numerous previous studies while revealing certain novel distinctions. Since the TyG index was proposed as a pragmatic surrogate for insulin resistance, multiple investigations have confirmed its association with cardiometabolic risk and adverse cardiovascular outcomes (17). Consistent with these observations, our study reveals that the insulin resistance component within CTI contributes substantially to liver synthetic dysfunction associated with prognosis in cardiac ICU populations. CRP represents a well-validated acute-phase marker correlated with myocardial injury severity and outcomes following acute coronary syndromes (18). Elevated CRP levels have been linked to decreased serum albumin, reflecting albumin’s characteristic behavior as a negative acute-phase protein (19). Our data similarly demonstrate a well-defined, independent negative correlation between CTI and both ALB and PA, with stronger effects observed in male patients and higher CTI strata, suggesting that integrating inflammatory and metabolic information provides superior discriminatory power compared to either parameter alone. The TBIL/ALB ratio is extensively utilized for hepatic function grading in chronic liver disease and oncology. In contrast, we observed no independent association between CTI and TBIL/ALB in the immediate post-MI ICU setting. While this finding may appear unexpected, the underlying mechanism is explicable. Research demonstrates that during acute inflammatory states, inflammatory markers primarily correlate negatively with negative acute-phase proteins (such as albumin and prealbumin), while their impact on bilirubin metabolism, though present, remains relatively limited (20–22). The relationship between TBIL/ALB and inflammation is comparatively restricted, largely because bilirubin possesses independent anti-inflammatory properties, and its fluctuations more accurately reflect hepatocyte functional status rather than direct inflammatory response products (23, 24). This selective association pattern suggests that CTI predominantly reflects the specific inhibition of hepatic protein synthesis and transport function by systemic inflammation and metabolic stress, rather than alterations in bilirubin metabolic pathways or hepatocyte injury patterns. During acute-phase responses, cytokine signaling redirects hepatic protein synthesis priorities, upregulating positive acute-phase proteins (such as CRP) while substantially suppressing negative acute-phase protein production (ALB, PA). Bilirubin clearance and metabolic pathways remain relatively stable in the short term and are less susceptible to direct modulation by inflammatory-metabolic signals (25, 26). Although previous cardiac surgery and critical care studies have documented post-operative liver dysfunction and hypoalbuminemia, to our knowledge, no investigation has specifically examined the relationship between a “combined inflammation-insulin resistance index” and early hepatic synthetic markers in MI ICU populations. This study addresses this gap by focusing on the critical biological and clinical window of the initial 24 h.

The selective association between CTI and ALB/PA observed in our study, with no corresponding relationship to TBIL/ALB, demonstrates clear biological mechanistic plausibility. This association pattern reflects the specific regulatory effects of acute inflammation and metabolic stress on hepatic protein synthesis function while relatively preserving the integrity of bilirubin metabolic pathways. The inflammation-driven hepatic protein synthesis “reprioritization” mechanism plays a central role in this process. Pro-inflammatory cytokines, particularly IL-6, IL-1β, and TNF-α, activate the JAK–STAT signaling pathway to reprogram hepatocyte protein synthesis patterns, upregulating transcription of positive acute-phase proteins (such as CRP and fibrinogen) while simultaneously downregulating gene expression of negative acute-phase proteins (ALB, PA/transthyretin) through inhibition of C/EBP-β and HNF-1α transcription factors (27, 28). Insulin resistance-mediated hepatic synthetic metabolic inhibition amplifies this effect through multiple molecular mechanisms. Impaired insulin signaling reflected by the TyG index leads to decreased PI3K/AKT/mTOR pathway activity, directly suppressing S6K1 and 4E-BP1 phosphorylation, thereby reducing protein translation initiation complex formation while limiting amino acid transporter activity (29, 30). This ultimately results in significantly decreased translation efficiency of albumin and prealbumin. Under conditions of cardiogenic shock or vasoactive drug support, splanchnic microcirculatory dysfunction activates hypoxia-inducible factor-1α (HIF-1α), altering hepatocyte metabolic priorities from anabolic processes toward glycolysis and survival signaling, further amplifying inflammation-metabolic synthetic inhibition (31). The lack of association between TBIL/ALB ratio and CTI stems from the relative independence of bilirubin metabolic pathways. Bilirubin production is primarily determined by red blood cell lifespan and hemoglobin degradation, remaining relatively constant during the acute phase (within 24 h). Hepatic bilirubin uptake relies mainly on organic anion-transporting polypeptide (OATP)-mediated carrier transport processes rather than protein synthesis pathways. The binding between bilirubin and albumin represents a reversible physicochemical process that does not require new protein synthesis (32, 33). Although alternative explanations such as dilutional hypoalbuminemia from fluid resuscitation, extravascular redistribution due to capillary leak, exogenous albumin administration, or short-term nutritional deficiency may exist, the synchronous and dose-dependent decline in both PA (half-life 2–3 days) and ALB (half-life 19–21 days) strongly supports a “synthesis inhibition-dominated” mechanism rather than mere distributional abnormalities (34). The negative findings for TBIL/ALB ratio further exclude widespread hepatocyte damage or cholestasis as primary driving factors, confirming the selective, specific regulation of hepatic protein synthesis function by inflammatory and metabolic signals (35). Sensitivity analyses revealed differential WBC adjustment effects across markers. CTI–ALB and CTI–PA associations remained significant after WBC adjustment, whereas CTI–TBIL/ALB was attenuated. This reflects distinct biological mechanisms: ALB and PA are negative acute-phase proteins specifically regulated by inflammatory-metabolic stress, while total bilirubin is influenced by WBC-related hemolysis, oxidative stress, and cholestasis (36). Thus, ALB and PA are more specific markers of CTI-associated hepatic dysfunction, while TBIL/ALB reflects general inflammatory burden.

Several limitations warrant acknowledgment. First, as a single-center retrospective study with a 24-h observation window, causal inference is limited. However, the substantial effect sizes, consistent dose–response relationships, comprehensive covariate adjustment, and multiple sensitivity analyses support the robustness of the observed associations. While the short timeframe precludes assessment of long-term outcomes, it precisely captures the critical period of acute post-operative inflammatory-metabolic response—the primary focus of our investigation. Second, pro-inflammatory cytokines (IL-6, TNF-α) were not routinely measured and could not be included as covariates. Since these cytokines lie on the mechanistic pathway linking CTI to hepatic dysfunction—mediating CRP induction and directly suppressing albumin and prealbumin synthesis—adjusting for them might paradoxically introduce overadjustment bias rather than improve confounder control. Third, although previous studies have documented post-operative liver dysfunction and hypoalbuminemia following cardiac surgery, no investigation has specifically examined the relationship between a combined inflammation-insulin resistance index and early hepatic synthetic markers in post-MI ICU populations. This represents a novel knowledge gap addressed by our study. To overcome these limitations, future research should prioritize prospective multicenter validation with larger, heterogeneous cohorts to enhance generalizability and enable causal inference. Serial measurements extending beyond 24 h would clarify the temporal evolution of CTI-hepatic associations and their prognostic implications. Comprehensive cytokine profiling (IL-6, TNF-*α*) combined with formal mediation analyses would quantify the relative contributions of inflammatory versus insulin resistance pathways. Additionally, incorporating direct measures of hepatic perfusion (e.g., indocyanine green clearance) and standardized perioperative data capture (anticoagulation, transfusion protocols) would reduce residual confounding and strengthen mechanistic understanding. Such prospective designs would facilitate translation of CTI into risk stratification tools and guide targeted therapeutic interventions in the acute post-MI period.

This study offers substantial clinical translational value. Based on routine laboratory parameters, CTI enables rapid assessment of hepatic protein synthetic dysfunction risk within the early post-myocardial infarction ICU period (within 24 h), providing valuable supplementation to traditional liver function monitoring. Patients with elevated CTI may benefit from individualized management approaches, including hemodynamic targets optimized for splanchnic perfusion, glycemic and lipid management strategies to mitigate insulin resistance, timely and adequate protein nutritional support, and early recognition of capillary leak-related protein losses. Translating these findings into evidence-based practice requires systematic follow-up investigations. Prospective multicenter validation studies incorporating dynamic ALB and PA monitoring are essential to define temporal coupling relationships and establish clinical decision thresholds. Additionally, intervention trials targeting the inflammation-insulin resistance axis should be designed to evaluate whether modulating CTI components through anti-inflammatory therapy, insulin sensitization, or precision splanchnic resuscitation strategies can improve hepatic protein synthetic trajectories and clinical outcomes. The practical implementation of CTI-guided care pathways could potentially enhance early identification of patients at risk for protein-calorie malnutrition and metabolic complications, facilitating more precise resource allocation in the intensive care setting. However, the optimal integration of CTI monitoring into existing critical care protocols requires further investigation to determine cost-effectiveness and impact on patient-centered outcomes.

Our data demonstrate that CTI captures clinically meaningful synthesis-centered hepatic vulnerability during the earliest post-MI ICU phase. While CTI does not serve as an independent diagnostic tool for liver failure, it provides actionable information beyond standard covariates and aligns with established biology linking acute-phase responses to insulin resistance. The observed associations between elevated CTI and decreased albumin and prealbumin levels reflect specific regulatory effects on hepatic protein synthesis rather than broader hepatocellular dysfunction, as evidenced by the absence of correlation with bilirubin metabolism markers. Following prospective validation and threshold calibration, CTI holds promise for integration into early post-MI ICU assessment protocols. Such implementation could guide precision supportive therapies aimed at preserving hepatic synthetic function, potentially optimizing patient recovery through targeted interventions addressing the inflammation-metabolic stress axis that underlies protein synthesis impairment in this vulnerable population.

## 5 Conclusion

In conclusion, this single-center retrospective study demonstrates that the CRP-TyG index (CTI) serves as a valuable early biomarker for hepatic synthetic dysfunction in post-myocardial infarction ICU patients within 24 h. Our findings reveal selective associations between elevated CTI and reduced albumin and prealbumin levels, while showing no correlation with TBIL/ALB ratio, suggesting specific inflammatory-metabolic mediated impairment of hepatic protein synthesis rather than global liver damage. The robust linear dose–response relationships observed across multiple analytical frameworks, combined with consistent findings in sensitivity analyses and mechanistic plausibility through inflammation-insulin resistance coupling pathways, support the clinical utility of CTI as an integrated assessment tool. These results provide novel insights into the pathophysiological interplay between systemic inflammation, metabolic dysregulation, and hepatic synthetic capacity in critically ill cardiac patients, offering potential for enhanced risk stratification and targeted therapeutic interventions to optimize patient outcomes in post-MI intensive care settings.

## Data Availability

The data analyzed in this study is subject to the following licenses/restrictions: the authors will make available the raw data supporting the conclusions of this article without reservation. Data can be obtained by contacting them via email. Requests to access these datasets should be directed to Jiao Bao, baojiaozi1984@163.com.
