# Supplementary material for: Association between CRP-TyG index and hepatic synthetic function in post-myocardial infarction ICU patients
Source: Front Med (Lausanne). 2025 Dec 11;12:1710430. doi: 10.3389/fmed.2025.1710430 (PMC12738846; doi:10.3389/fmed.2025.1710430)
Supplement: Supplementary file 1 [file Supplementary_file_1.docx]

Supplementary-table 1 Covariate collinearity analysis

| **Variable** | **VIF** | **CI_lower** | **CI_upper** |
| --- | --- | --- | --- |
| Cti | 1.349 | 1.283 | 1.681 |
| Age | 1.776 | 1.661 | 2.324 |
| Sex | 2.27 | 2.136 | 2.951 |
| Status | 1.481 | 1.35 | 2 |
| Smk | 1.964 | 1.804 | 2.601 |
| Alcohol | 1.294 | 1.249 | 1.612 |
| Hypertension | 1.346 | 1.297 | 1.668 |
| DM | 1.374 | 1.335 | 1.723 |
| CKD | 1.428 | 1.301 | 2.04 |
| CHF | 1.207 | 1.2 | 1.515 |
| COPD | 1.301 | 1.244 | 1.596 |
| CVD | 1.277 | 1.244 | 1.598 |
| Tumor | 1.073 | 1.086 | 1.298 |
| Sepsis | 1.43 | 1.394 | 1.788 |
| CRRT | 1.86 | 1.605 | 2.773 |
| Therapy | 1.115 | 1.117 | 1.289 |
| RBC | 1.609 | 1.533 | 2.12 |
| TC | 2.382 | 1.746 | 5.808 |
| LDL | 2.187 | 1.685 | 4.946 |
| ALT | 2.448 | 2.235 | 3.63 |
| AST | 2.657 | 2.339 | 3.885 |
| PT | 1.444 | 1.391 | 2.666 |
| BUN | 3.328 | 2.314 | 5.859 |
| pScr_7d | 2.735 | 2.268 | 4.681 |
| baseline_Scr | 3.348 | 2.246 | 5.955 |

Supplementary-table2 Associaition between the CTI and ALB/PA/Ratio_TbilALB cut_off by Logistic model.

| **Characteristic** | **ALB-Crude** | | **ALB-Adjusted** | |
| --- | --- | --- | --- | --- |
|  | **OR(95%CI)** | ***P*** | **OR(95%CI)** | ***P*** |
| CTI (per 1 unit) | 2.06(1.41-3.06) | <0.001* | 2.03(1.18-3.59) | 0.012* |
| CTI quartile |  |  |  |  |
| Q1 | ref | | ref | |
| Q2 | 0.57(0.19-1.64) | 0.307 | 0.53(0.12-2.13) | 0.386 |
| Q3 | 1.8(0.77-4.43) | 0.184 | 1.56(0.48-5.22) | 0.465 |
| Q4 | 3.72(1.68-8.79) | 0.002* | 4.25(1.37-14.06) | 0.014* |
| P for trends |  | <0.001* |  | 0.040* |
| **Characteristic** | **PA-Crude** | | **PA-Adjusted** | |
|  | **OR(95%CI)** | ***P*** | **OR(95%CI)** | ***P*** |
| CTI (per 1 unit) | 1.84(1.26-2.74) | 0.002* | 2.88(1.74-4.94) | <0.001* |
| CTI quartile |  |  |  |  |
| Q1 | ref | | ref | |
| Q2 | 2.02(0.72-6.17) | 0.192 | 3.1(0.89-11.71) | 0.081 |
| Q3 | 2.47(0.91-7.40) | 0.086 | 3.99(1.22-14.38) | 0.026* |
| Q4 | 5.85(2.36-16.76) | <0.001* | 13.32(4.34-46.81) | <0.001* |
| P for trends |  | <0.001* |  | <0.001* |
| **Characteristic** | **TBIL/ABL-Crude** | | **TBIL/ABL-Adjusted** | |
|  | **OR(95%CI)** | ***P*** | **OR(95%CI)** | ***P*** |
| CTI (per 1 unit) | 1.26(0.68-2.35) | 0.47 | 1.79(0.71-4.74) | 0.225 |
| CTI quartile |  |  |  |  |
| Q1 | ref | | ref | |
| Q2 | 1.01(0.23-4.45) | 0.984 | 1.47(0.25-8.94) | 0.667 |
| Q3 | 0.75(0.14-3.52) | 0.713 | 0.68(0.09-4.26) | 0.677 |
| Q4 | 1.55(0.42-6.28) | 0.515 | 4.02(0.58-31.32) | 0.162 |
| P for trends |  | 0.578 |  | 0.384 |

****P*** <0.05; ALB <35 indicates impaired protein synthesis, resulting in hypoproteinemia; PA <150 is abnormal; TBil/Alb >0.6 is abnormal.

Adjusted for Age, Gender, Status, Smoking, Alcohol, Hypertension, Diabetes Mellitus (DM), Chronic Kidney Disease (CKD), Congestive Heart Failure (CHF), Chronic Obstructive Pulmonary Disease (COPD), Cardiovascular Disease (CVD), Tumor, Sepsis, Continuous Renal Replacement Therapy (CRRT), Vasopressor Therapy, Red Blood Cell Count (RBC), Total Cholesterol (TC), Low-Density Lipoprotein (LDL), Alanine Aminotransferase (ALT), Aspartate Aminotransferase (AST), Prothrombin Time (PT), Blood Urea Nitrogen (BUN), Serum Creatinine Before ICU (Scr Before ICU), and Maximum Serum Creatinine After ICU (Max Scr After ICU).

Supplementary-table3 Associaition between the CTI and ALB/PA/Ratio_TbilALB by OLS model.

| **Characteristic** | **ALB-Crude** | | **ALB-Adjusted** | | **ALB-Adjusted#** | |
| --- | --- | --- | --- | --- | --- | --- |
|  | **β(SE)** | ***P*** | **β(SE)** | ***P*** | **β(SE)** | ***P*** |
| CTI (per 1 unit) | -1.21 (0.35) | <0.001* | -1.23 (0.32) | <0.001* | 0.07 (0.03) | 0.015 |
| CTI quartile |  |  |  |  |  |  |
| Q1 | ref | | ref | |  | |
| Q2 | -0.14 (0.79) | 0.863 | -0.30 (0.68) | 0.66 | -0.04 (0.06) | 0.512 |
| Q3 | -1.97 (0.79) | 0.013* | -1.81 (0.67) | 0.008* | 0.04 (0.06) | 0.465 |
| Q4 | -2.30 (0.78) | 0.004* | -2.19 (0.71) | 0.002* | 0.16 (0.06) | 0.012* |
| P for trends |  | <0.001* |  | <0.001* |  | 0.006* |
| **Characteristic** | **PA-Crude** | | **PA-Adjusted** | | **PA-Adjusted#** | |
|  | **β(SE)** | ***P*** | **β(SE)** | ***P*** | **β(SE)** | ***P*** |
| CTI (per 1 unit) | -22.67 (5.01) | <0.001* | -26.35 (5.07) | <0.001* | 0.13 (0.03) | <0.001* |
| CTI quartile |  |  |  |  |  |  |
| Q1 | ref | | ref | |  | |
| Q2 | -10.01 (11.18) | 0.371 | -8.56 (10.48) | 0.415 | 0.11 (0.06) | 0.082 |
| Q3 | -27.56 (11.18) | 0.014* | -29.48 (10.46) | 0.005* | 0.14 (0.07) | 0.034* |
| Q4 | -53.74 (11.14) | <0.001* | -56.05 (10.98) | <0.001* | 0.33 (0.07) | <0.001* |
| P for trends |  | <0.001* |  | <0.001* |  | <0.001* |
| **Characteristic** | **TBIL/ABL-Crude** | | **TBIL/ABL-Adjusted** | | **TBIL/ABL-Adjusted#** | |
|  | **β(SE)** | ***P*** | **β(SE)** | ***P*** | **β(SE)** | ***P*** |
| CTI (per 1 unit) | 0.02 (0.01) | 0.219 | 0.02 (0.01) | 0.135 | 0.01 (0.02) | 0.595 |
| CTI quartile |  |  |  |  |  |  |
| Q1 | ref | | ref | |  | |
| Q2 | 0.02 (0.03) | 0.571 | -0.00 (0.03) | 0.995 | -0.02 (0.04) | 0.664 |
| Q3 | 0.03 (0.03) | 0.263 | 0.03 (0.03) | 0.324 | -0.03 (0.04) | 0.507 |
| Q4 | 0.04 (0.03) | 0.179 | 0.04 (0.03) | 0.132 | 0.02 (0.05) | 0.633 |
| P for trends |  | 0.146 |  | 0.081 |  | 0.718 |

****P*** <0.05; ALB <35 indicates impaired protein synthesis, resulting in hypoproteinemia; PA <150 is abnormal; TBil/Alb >0.6 is abnormal.

Adjusted for Age, Gender, Status, Smoking, Alcohol, Hypertension, Diabetes Mellitus (DM), Chronic Kidney Disease (CKD), Congestive Heart Failure (CHF), Chronic Obstructive Pulmonary Disease (COPD), Cardiovascular Disease (CVD), Tumor, Sepsis, Continuous Renal Replacement Therapy (CRRT), Vasopressor Therapy, Red Blood Cell Count (RBC), Total Cholesterol (TC), Low-Density Lipoprotein (LDL), Alanine Aminotransferase (ALT), Aspartate Aminotransferase (AST), Prothrombin Time (PT), Blood Urea Nitrogen (BUN), Serum Creatinine Before ICU (Scr Before ICU), and Maximum Serum Creatinine After ICU (Max Scr After ICU).

#Further adjusted by WBC

Supplementary-table4 Associaition between the CTI and ALB cut_off by OLS model among thoes with status=0/ Pressor Therapy/ CRRT Therapy.

| **Characteristic** | **ALB-Crude** | | **ALB-Adjusted***#* | | |
| --- | --- | --- | --- | --- | --- |
|  | **β(SE)** | ***P*** | **β(SE)** | ***P*** | **Adjusted P†** |
| **Status** |  |  |  |  |  |
| CTI (per 1 unit) | -0.72 (0.37) | 0.053 | -0.99 (0.33) | 0.003 | 0.009* |
| CTI quartile |  |  |  |  |  |
| Q1 | ref |  | ref |  |  |
| Q2 | -0.21 (0.78) | 0.787 | -0.50 (0.68) | 0.456 | 1 |
| Q3 | -1.71 (0.77) | 0.028* | -1.74 (0.68) | 0.012 | 0.036* |
| Q4 | -1.27 (0.81) | 0.115 | -1.80 (0.72) | 0.013 | 0.039* |
| P for trends |  | 0.032* |  | 0.003 | 0.009* |
| **Pressor Therapy** |  |  |  |  |  |
| CTI (per 1 unit) | -1.10 (0.37) | 0.003* | -1.23 (0.35) | <0.001 | <0.001* |
| CTI quartile |  |  |  |  |  |
| Q1 | ref |  | ref |  |  |
| Q2 | 0.24 (0.86) | 0.777 | 0.06 (0.74) | 0.937 | 1 |
| Q3 | -1.59 (0.86) | 0.066 | -1.47 (0.74) | 0.048 | 0.144 |
| Q4 | -1.79 (0.85) | 0.035* | -1.87 (0.77) | 0.016 | 0.048* |
| P for trends |  | 0.008* |  | 0.003 | 0.009* |
| **CRRT Therapy** |  |  |  |  |  |
| CTI (per 1 unit) | -1.13 (0.37) | 0.002* | -1.19 (0.33) | <0.001 | <0.001* |
| CTI quartile |  |  |  |  |  |
| Q1 | ref |  | ref |  |  |
| Q2 | -0.00 (0.84) | 1 | -0.37 (0.70) | 0.593 | 1 |
| Q3 | -1.76 (0.84) | 0.037* | -1.45 (0.71) | 0.041 | 0.123 |
| Q4 | -2.13 (0.83) | 0.011* | -2.30 (0.73) | 0.002 | 0.006* |
| P for trends |  | 0.002* |  | <0.001 | <0.001* |

****P*** <0.05;

*#*Adjusted by gender, status, smoking, alcohol, Hypertension, DM, CKD, CHF, COPD, CVD, tumor, Sepsis, RBC, TC, LDL, ALT, AST, PT, BUN, Scr before ICU, max Scr after ICU.

**†**Bonferroni correction

Supplementary-table5 Associaition between the CTI and PA cut_off by OLS model among thoes with status=0/ Pressor Therapy/ CRRT Therapy.

| **Characteristic** | **PA-Crude** | | **PA-Adjusted***#* | | |
| --- | --- | --- | --- | --- | --- |
|  | **β(SE)** | ***P*** | **β(SE)** | ***P*** | **Adjusted P†** |
| **Status** |  |  |  |  |  |
| CTI (per 1 unit) | -19.35 (5.56) | <0.001* | -23.21 (5.31) | <0.001* | <0.001* |
| CTI quartile |  |  |  |  |  |
| Q1 | ref |  | ref |  |  |
| Q2 | -12.33 (11.67) | 0.292 | -15.00 (10.65) | 0.16 | <0.001* |
| Q3 | -23.66 (11.62) | 0.043* | -25.83 (10.75) | 0.017* | 0.051 |
| Q4 | -46.91 (12.10) | <0.001* | -54.58 (11.37) | <0.001* | <0.001* |
| P for trends |  | <0.001* |  | <0.001* | <0.001* |
| **Pressor Therapy** |  |  |  |  |  |
| CTI (per 1 unit) | -25.05 (5.32) | <0.001* | -28.94 (5.56) | <0.001* | <0.001* |
| CTI quartile |  |  |  |  |  |
| Q1 | ref |  | ref |  |  |
| Q2 | -7.36 (12.11) | 0.544 | -6.33 (11.82) | 0.593 | 1 |
| Q3 | -28.63 (12.16) | 0.019* | -28.73 (11.80) | 0.016* | 0.048* |
| Q4 | -55.75 (11.97) | <0.001* | -59.58 (12.20) | <0.001* | <0.001* |
| P for trends |  | <0.001* |  | <0.001* | <0.001* |
| **CRRT Therapy** |  |  |  |  |  |
| CTI (per 1 unit) | -22.85 (4.82) | <0.001* | -25.69 (5.03) | <0.001* | <0.001* |
| CTI quartile |  |  |  |  |  |
| Q1 | ref |  | ref |  |  |
| Q2 | -4.90 (10.85) | 0.651 | -9.76 (10.39) | 0.348 | 1 |
| Q3 | -23.91 (10.93) | 0.030* | -28.20 (10.54) | 0.008* | 0.024* |
| Q4 | -52.00 (10.76) | <0.001* | -55.80 (10.90) | <0.001* | <0.001* |
| P for trends |  | <0.001* |  | <0.001* | <0.001* |

****P*** <0.05;

*#*Adjusted by gender, status, smoking, alcohol, Hypertension, DM, CKD, CHF, COPD, CVD, tumor, Sepsis, RBC, TC, LDL, ALT, AST, PT, BUN, Scr before ICU, max Scr after ICU.

**†**Bonferroni correction

Supplementary-table6 Associaition between the CTI and Tbil/ALB cut_off by OLS model among thoes with status=0/ Pressor Therapy/ CRRT Therapy.

| **Characteristic** | **TBIL/ABL-Crude** | | **TBIL/ABL-Adjusted***#* | | |
| --- | --- | --- | --- | --- | --- |
|  | **β(SE)** | ***P*** | **β(SE)** | ***P*** | **Adjusted P†** |
| **Status** |  |  |  |  |  |
| CTI (per 1 unit) | 0.01 (0.01) | 0.394 | 0.01 (0.01) | 0.303 | 0.909 |
| CTI quartile |  |  |  |  |  |
| Q1 | ref |  | ref |  |  |
| Q2 | 0.02 (0.03) | 0.388 | 0.00 (0.03) | 0.867 | 1 |
| Q3 | 0.03 (0.03) | 0.255 | 0.03 (0.03) | 0.248 | 0.744 |
| Q4 | 0.02 (0.03) | 0.535 | 0.02 (0.03) | 0.399 | 1 |
| P for trends |  | 0.452 |  | 0.254 | 0.762 |
| **Pressor Therapy** |  |  |  |  |  |
| CTI (per 1 unit) | 0.01 (0.01) | 0.311 | 0.02 (0.01) | 0.223 | 0.669 |
| CTI quartile |  |  |  |  |  |
| Q1 | ref |  | ref |  |  |
| Q2 | 0.03 (0.03) | 0.368 | 0.01 (0.03) | 0.72 | 1 |
| Q3 | 0.02 (0.03) | 0.444 | 0.02 (0.03) | 0.439 | 1 |
| Q4 | 0.04 (0.03) | 0.158 | 0.04 (0.03) | 0.146 | 0.438 |
| P for trends |  | 0.192 |  | 0.129 | 0.387 |
| **CRRT Therapy** |  |  |  |  |  |
| CTI (per 1 unit) | 0.01 (0.01) | 0.319 | 0.02 (0.01) | 0.212 | 0.636 |
| CTI quartile |  |  |  |  |  |
| Q1 | ref |  | ref |  |  |
| Q2 | 0.01 (0.03) | 0.731 | -0.00 (0.03) | 0.875 | 1 |
| Q3 | 0.01 (0.03) | 0.629 | 0.02 (0.03) | 0.559 | 1 |
| Q4 | 0.03 (0.03) | 0.245 | 0.04 (0.03) | 0.184 | 0.552 |
| P for trends |  | 0.248 |  | 0.136 | 0.408 |

****P*** <0.05;

*#*Adjusted by gender, status, smoking, alcohol, Hypertension, DM, CKD, CHF, COPD, CVD, tumor, Sepsis, RBC, TC, LDL, ALT, AST, PT, BUN, Scr before ICU, max Scr after ICU.

**†**Bonferroni correction
